# ASGDB: a specialised genomic resource for interpreting *Anopheles sinensis* insecticide resistance

**DOI:** 10.1186/s13071-017-2584-8

**Published:** 2018-01-10

**Authors:** Dan Zhou, Yang Xu, Cheng Zhang, Meng-Xue Hu, Yun Huang, Yan Sun, Lei Ma, Bo Shen, Chang-Liang Zhu

**Affiliations:** Department of Pathogen Biology, Nanjing Medical University, Nanjing, Jiangsu 210029 People’s Republic of China

**Keywords:** Database, *Anopheles sinensis*, Insecticide resistance

## Abstract

**Background:**

*Anopheles sinensis* is an important malaria vector in Southeast Asia. The widespread emergence of insecticide resistance in this mosquito species poses a serious threat to the efficacy of malaria control measures, particularly in China. Recently, the whole-genome sequencing and *de novo* assembly of *An. sinensis* (China strain) has been finished. A series of insecticide-resistant studies in *An. sinensis* have also been reported. There is a growing need to integrate these valuable data to provide a comprehensive database for further studies on insecticide-resistant management of *An. sinensis*.

**Results:**

A bioinformatics database named *An. sinensis* genome database (ASGDB) was built. In addition to being a searchable database of published *An. sinensis* genome sequences and annotation, ASGDB provides in-depth analytical platforms for further understanding of the genomic and genetic data, including visualization of genomic data, orthologous relationship analysis, GO analysis, pathway analysis, expression analysis and resistance-related gene analysis. Moreover, ASGDB provides a panoramic view of insecticide resistance studies in *An. sinensis* in China. In total, 551 insecticide-resistant phenotypic and genotypic reports on *An. sinensis* distributed in Chinese malaria-endemic areas since the mid-1980s have been collected, manually edited in the same format and integrated into OpenLayers map-based interface, which allows the international community to assess and exploit the high volume of scattered data much easier. The database has been given the URL: http://www.asgdb.org/.

**Conclusions:**

ASGDB was built to help users mine data from the genome sequence of *An. sinensis* easily and effectively, especially with its advantages in insecticide resistance surveillance and control.

**Electronic supplementary material:**

The online version of this article (10.1186/s13071-017-2584-8) contains supplementary material, which is available to authorized users.

## Background

Malaria remains one of the most important mosquito-borne infectious parasitic diseases in tropical and subtropical areas [1, 2]. According to the World Health Organization (WHO), an estimated 212 million new cases and 429,000 deaths associated with malaria were reported in 2015 [3]. Malaria has been extensively endemic in China for more than 4000 years, and several large-scale outbreaks of malaria have occurred in Chinese history [4, 5]. Through sustained governmental and international organizational efforts in different control phases, malaria cases were dramatically decreased from approximately 30 million in 1949 to 7855 in 2010 [6, 7]. In the same year, the National Malaria Elimination Programme (2010–2020) was initiated [8]. The mid-term evaluation of this programme was revealed that malaria declined to 3078 cases in 2014, including 25 deaths [9, 10]. Although remarkable progress in malaria control has been achieved, challenges still exist on the path towards malaria elimination. For example, there were continued unpredictable outbreaks of *vivax* malaria in the central region of China along the Huang-Huai River since 2001 [5]. Moreover, the increasing number of imported malaria cases causes new threats to malaria elimination [11].

Without an efficient vaccine, vector control is considered as a key intervention to control and possibly eliminate malaria. Malaria is transmitted by mosquitoes of the genus *Anopheles*. In Southeast Asia, more than 30 *Anopheles* species occur in the domestic environment [12]. *Anopheles dirus*, *An. minimus* and *An. epiroticus* are considered the major vectors which contribute significantly to malaria transmission [13]. In addition, several other *Anopheles* species may also be locally involved [13]. In China, there are four main malaria vectors, *An. sinensis*, *An. dirus*, *An. anthropophagus* (synonymy with *An. lesteri*) and *An. minimus* [14]. Although *An. sinensis* shows zoophilic and exophilic behaviours, it is still considered the most dominant natural vector of *Plasmodium vivax* in China due to several factors: (i) *An. sinensis* is the most widespread species distributed in most parts of China, from the northeast (Liaoning Province) to southwest (Yunnan Province); (ii) *An. sinensis* is susceptible to *P. vivax* and the ability of *An. sinensis* to transmit *P. vivax* has been obviously enhanced in China [15]; (iii) *An. sinensis* can shift from zoophilic (first option) to anthropophilic (second option) behaviour in areas where the number of cattle and pigs decreased [15]. In particular, *An. sinensis* is considered as the most frequently and widely distributed cause of recurring malaria in the central part of China and may represent a major risk for malaria elimination [16, 17].

Vector control is considered as a key intervention to control and possibly eliminate malaria, especially the long-lasting insecticide-treated bednets (LLINs) and indoor residual spraying (IRS), which were used mainly in southern China [18, 19]. Pyrethroids are currently the only class of insecticide approved by the WHO for bednet impregnation, and they are also used for IRS [20]. However, studies have shown that the level of resistance was proportional to the insecticide selection pressure [21]. Thus, constant and intensive insecticide exposure created a high selection pressure and favoured the development of resistance [21–23]. Phenotypic resistance to DDT and pyrethroids in *An. sinensis* could be found throughout China, which has placed the current national efforts of malaria elimination at risk [24]. Resistance to various classes of insecticides in *An. sinensis* has also been reported in South Korea [25].

WHO launched the Global Plan for Insecticide Resistance Management in malaria vectors (GPIRM) in May 2012 [26]. This plan is a collective strategy aimed at collecting baseline information on insecticide resistance at the global scale and further understanding the molecular mechanism of insecticide resistance. Insecticide resistance is a complex phenotype of polygenic inheritance, which is mediated through the interaction of multiple genes. The complete genome of *An. sinensis* (China strain) has been sequenced and fully annotated recently, which provides a credible starting point to determine the roles that multiple genes play in insecticide resistance. Moreover, a series of insecticide resistance-related studies for *An. sinensis* in China have been conducted since the last century, which undoubtedly provides us with massive and openly accessible information, from the standard WHO susceptibility tests to resistance-related molecules characterization [27–30]. However, these data from different researchers are scattered in various publications. Researchers have to spend a tremendous amount of time to acquire information of interest and to evaluate the current status of insecticide resistance systematically. To host the fast-growing amount of genomic data and aid ongoing insecticide resistance research, a highly integrated information platform is needed for storage, retrieval, visualization and analysis of the *An. sinensis* genomic data and phenotypic data.

Some *An. sinensis* genomic or genetic data sets are available on NCBI (https://www.ncbi.nlm.nih.gov/) [31] or VectorBase (https://www.vectorbase.org/) [32]. NCBI is a database meant for general use and is not specifically designed for people working on vectors and insecticide resistance. Although VectorBase is specially designed for meeting the need for most of the community working on vectors, the accumulated genomic and phenotypic information of *An. sinensis* (China strain) is quite limited. At present, the genomic or genetic information of *An. sinensis* (China strain) in VectorBase mainly focuses on gene sequence, protein information and gene ontology (GO) analysis, which falls short of what will be needed. In this study, we developed an integrated *An. sinensis* genome database (ASGDB; database homepage: http://www.asgdb.org/). Besides providing the published sequences and annotation, ASGDB can also be used as an online analytical platform for visualization of genomic data, orthologous relationship analysis, GO analysis, Kyoto Encyclopedia of Genes and Genomes (KEGG) pathway analysis, expression analysis and resistance-related gene analysis. ASGDB also aims to become an important repository for insecticide resistance data for *An. sinensis* in China. Although there are some databases that produce maps of phenotypic insecticide resistance data in malaria vectors (including *An. sinensis*) worldwide such as IR Mapper (http://www.irmapper.com) or VectorBase (https://www.vectorbase.org/popbio), the information of insecticide resistance data for *An. sinensis* in China is limited. One important reason is much valuable information reported in the Chinese medical literature may not be accessible to the malaria research community outside of China because of language barriers. We have collected and processed 551 insecticide-resistant phenotypic and genotypic reports from English or Chinese literature from the mid-1980s or unpublished data provided by the public voluntarily. These data were manually edited in the same format and presented comprehensively and systematically in OpenLayers map-based interface. Users can easily track the dynamics and evolution of insecticide-resistant information in *An. sinensis* temporally and spatially.

## Methods

### Data collection

The complete genome sequence and annotation of *An. sinensis* was downloaded from the National Center for Biotechnology Information (NCBI) (http://www.ncbi.nlm.nih.gov/bioproject/PRJNA209295). The orthology relationships between the protein-coding gene sets from the different genomes were generated by the Ensembl Gene Tree method [33]. GO IDs for each gene were obtained from the corresponding InterPro entry. Each GO term is hyperlinked to AmiGO 2 browser at the GO website for details [34]. KEGG was used to perform pathway enrichment analysis of all genes [35]. *Anopheles sinensis* transcriptome data were downloaded from NCBI (BioProject accession numbers: PRJNA293400 and PRJNA339810). In a previous study, the field population of *An. sinensis* was collected in the field in Shifosi (N29.95, E115.62) town of Hubei Province in 2011. After 2 to 3-days post adult emergence, male adult and non-blood female adult mosquitoes were morphological identified and classified as the male-stain and female-stain, respectively. The other parts of non-blood female adult mosquitoes were phenotyped for susceptibility to 0.05% deltamethrin using the standard WHO tube susceptibility bioassay. The mosquitoes which knocked down after one-hour exposure were classified as deltamethrin-susceptible strain (DS-strain) and those survived after the 24-h recovery period were classified as the deltamethrin-resistant strain (DR-strain). Four libraries (male-stain, female-stain, DS-strain and DR-strain) were constructed to provide transcriptomic data to assess the assembly quality of the *An. sinensis* genome [36]. The differential gene expression levels (*P* < 0.05) between female-stain and male-stain or DS-strain and DR-strain were integrated into each gene’s basic information. The information for cytochrome P450 (P450), glutathione S-transferase (*gst*), choline/carboxylesterase (*cce*) and cuticular protein (*cp*) genes in several mosquito species and *Drosophila melanogaster* were mainly collected from relevant published papers [37–44]. The insecticide resistance data of *An. sinensis* in China was searched and analysed manually. Most of these data were collected from research articles published in PubMed (http://www.ncbi.nlm.nih.gov/pubmed/), CNKI (http://www.cnki.net/), Wanfang (http://www.wanfangdata.com.cn/) and VIP (http://www.cqvip.com/) since 1988. A comprehensive search of related publications was made by using the following search terms: “*Anopheles sinensis*” AND (“China” or the name of Chinese provinces/ cities/ counties/ autonomous regions/ municipality) AND (“insecticide” or the names of insecticides (shown in Additional file 1: Table S1) or “*kdr*” or “knockdown” or “voltage-gated sodium channel” or “detoxification” or “P450” or “*gst*” or “glutathione-S-transferase” or “*cce*” or “choline/carboxylesterase” or “cuticular”). The full text of each eligible publication was read, and detailed information in the study was extracted and refined manually, including collection details, insecticide resistance monitoring information and potential insecticide resistance mechanisms. The insecticide resistance phenotypic and genotypic data: through deep mining of published papers, 538 insecticide-resistant related events in China were integrated.The vast majority of the events (72%) were collected from Chinese journals. The most common journals were “Journal of Medical Pest Control” (19%, Chinese journal) and “Chinese Journal of Vector Biology and Control” (18%, Chinese journal) after the search. The insecticide resistance phenotypes were determined according to the standard WHO tube bioassay [45], and about 35% of the events lack insecticide resistance phenotypic information.

### System construction

**Fig. 1:**
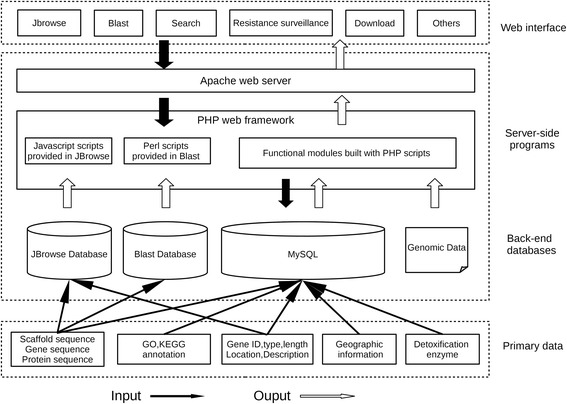
Overview of the ASGDB architecture

ASGDB database was developed on an Apache HTTP server in a Linux (CentOS 6.5) operating system. The web pages were written using PHP, HTML language, Cascading Style Sheets (CSS) and JavaScript. Custom Perl scripts were used to make the database user-friendly with a good interaction interface. The Apache server handles queries from web clients through PHP scripts to perform searches. In the back-end part, all of the data in ASGDB were stored and managed in MySQL relational databases. The JBrowse genome browser is a combination of database and interactive web pages for manipulating and visualizing genes with large-scale sequence datasets [46, 47]. This next generation AJAX-based genome browser, built with JavaScript and HTML5, enables fast and smooth animated genome navigation over the web. ViroBLAST has also been integrated into the ASGDB system [48]. The interface of “Resistance surveillance” utilizes OpenLayers 3 (high-performance, feature-packed web mapping library) to visualize the data. An overview of the ASGDB architecture is given in Fig. 1. The website has been tested on multiple platforms (Linux, Windows and Mac OS) with different web browsers (Firefox, Chrome, IE and Safari). The resistance surveillance map was not compatible with Firefox. We recommend using IE, Chrome, Opera or Safari to open the webpage of the insecticide-resistant map.

## Results

### Data content

There are three types of data included in ASGDB. Basic data: 19,317 putative protein-coding genes and 2425 non-coding RNAs predicted from 9597 *An. sinensis* assembled scaffolds are obtained. For all protein-coding genes, 1424 GO terms, 221 known pathways and 58,207 orthologs are displayed in ASGDB [36]. ASGDB also contains 4047 differentially expressed genes under different conditions from transcriptomic studies, including deltamethrin-resistant/ deltamethrin-susceptible and female/male [36]. Insecticide resistance-related gene data: ASGDB provides the identification and classification information of 93 cytochrome P450s (P450s), 31 glutathione S-transferases (*gst*s), 50 choline/carboxylesterases (*cce*s) and 238 cuticular proteins from *An. sinensis*. Related information of these gene families in other species is also provided for comparison. The database also contains 13 unpublished datasets provided by Dr Chang (Bengbu Medical College). Altogether, 551 insecticide-resistant related events are stored and managed in ASGDB, and a user-friendly web interface was developed to help users search and use these data. A summary of data content in ASGDB is shown in Table 1.

**Table 1:** Summary of ASGDB content

Data type	Data statistics
Gene	19,317
None coding RNA	2425
MicroRNAs	56
tRNAs	352
rRNA	2017
GO	1424
KEGG	221
Ortholog	58,207
Differentially expressed genes	4047
Female/Male	1388
Deltamethrin-resistant/ Deltamethrin-susceptible	2659
Insecticide-resistant related events	551
Published articles	538
Unpublished data	13

### Web interface and homepage

ASGDB interface provides direct links to eight individual pages including Home, JBrowse, Search, Download, Resistance-related gene, Resistance surveillance, Contact and Tutorial. All the links are clickable icons. A left mouse click on the icon leads to the respective page.

A brief introduction to ASGDB and photographs of *An. sinensis* taken at different life-cycle stages (egg, larva, pupa and adult) are included at the top part of the homepage. Below these, the latest publications related to *An. sinensis* are displayed. A link to the list of all relevant publications is also provided, allowing users acquire information of interest quickly and easily. “Resistance surveillance map” provides a map-based interface to access insecticide resistance-related data. All the collection sites are spotted with red pompons on the map. If users are interested in this content, they can click on the map and will be taken to a new page to acquire more detailed information. “Visitor Statistics” shows the number of visitors to the website every week.

### JBrowse

Here, we use the JBrowse genome browser to visualize the whole draft genome sequence of *An. sinensis.* The page is divided into two parts. The relatively small panel on the left is a list of different types of factors, on which the icons, from top to bottom, are: “GC content”, “gene”, “exon”, “miRNA”, “rRNA”, “tRNA” and “reference sequence”. All these icons for each scaffold can be tracked to the detailed view. The larger window on the right is the tracks display region. Each track can be turned off by clicking the “cross” in front of the title, which allows users to hide unwanted information for a better user experience. Dragging the tracks up or down can change the positions of tracks to display datasets of interest at the top for convenience. JBrowse also provides efficient panning and zooming of a genomic region in the genome via embedded navigation buttons. With the help of these important and efficient visualization modules, users can easily browse and search on a large scale in a graphic interface.

Users can search for scaffolds to locate regions on the *An. sinensis* genome. These scaffolds are freely selected from the drop-down menu. If the users are interested in a particular region on the scaffold, they can also enter the starting and terminal positions of the region to retrieve detailed information. In the genomic view, rectangular frames with directionality represent the corresponding genes or ncRNAs from the positive or negative strand. A single click on the frame will open an information table, which provides detailed information such as annotation, location, GO, KEGG and sequences. The sequence data for the selected gene can be downloaded in the same page as FASTA files. Other people can see the same region of the *An. sinensis* genome and the collection of open tracks on their screen when the visible URL (accessible either via the browser address bar or the “Share” button) is shared.

### Search

ASGDB affords a user-friendly search engine to make it easy to reach specific genes of interest. There are two sub-categories in the search part: simple search and BLAST search. To browse different types of genetic factors, a simple search can be performed using the following parameters: (i) NCBI or ASGDB accession numbers; (ii) Gene name or symbol; (iii) GO ID or GO term; and (iv) KEGG ID or KEGG annotation. Users can enter these parameters to obtain specific gene information from ASGDB, and fuzzy queries are supported. All the matched genes will be linked in the search job when more than one gene is matched with the input keyword. The BLAST search allows searching of genes using the ViroBLAST [48]. Users perform similarity searches against each type of sequences using various BLAST search forms (BLASTn, BLASTp, BLASTx, tBLASTn and tBLASTx). The reference database used for BLAST is all nucleic acid and amino acid sequences of *An. sinensis*. Users can enter nucleotide or protein query sequences or upload a local sequence file in the FASTA format to search against the reference database. The BLAST search tool allows users to set their favourite parameters, such as threshold, Word size, etc., in advanced search.

**Fig. 2:**
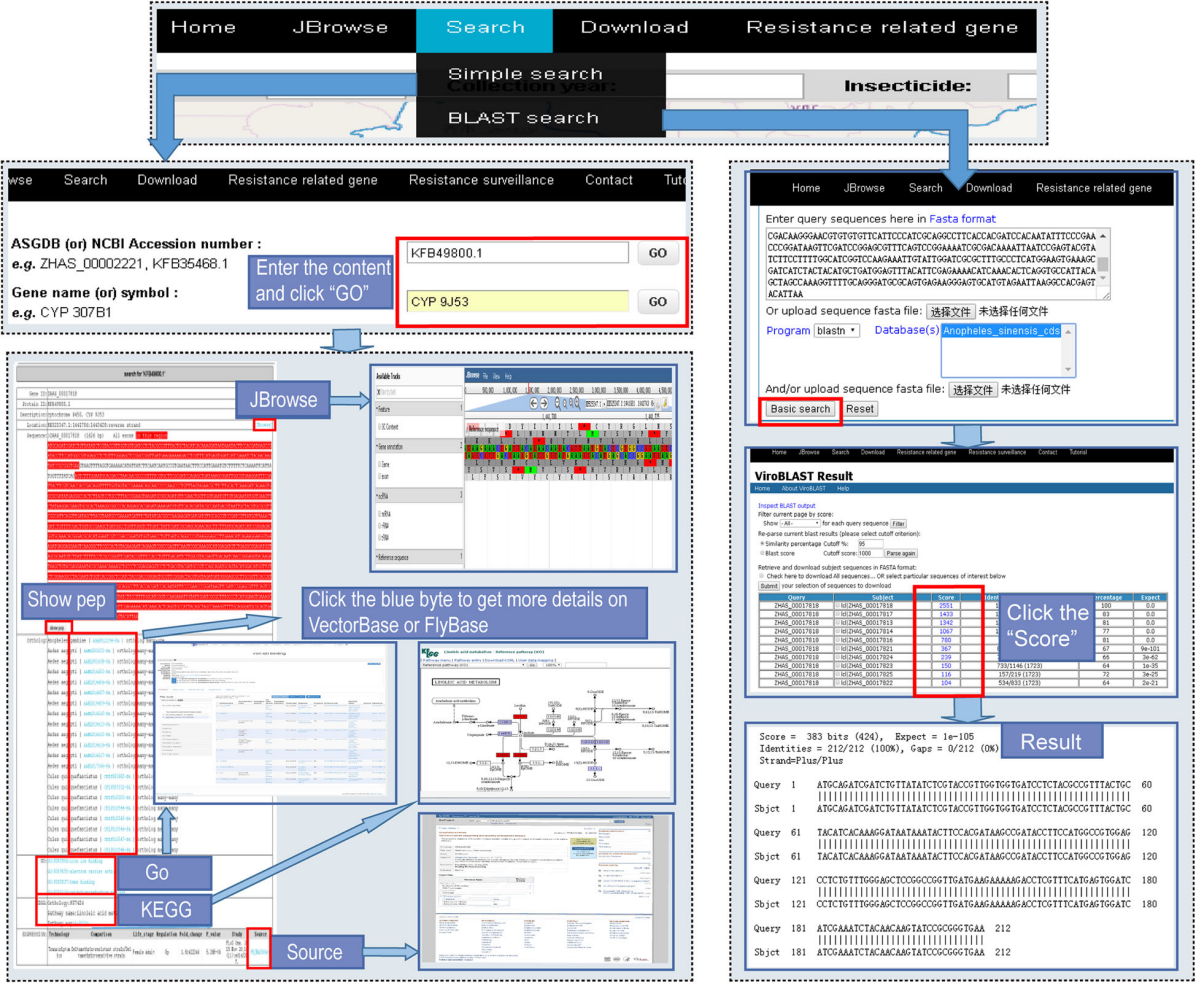
Screenshot showing the application of ASGDB for searching information

This multi-functional search module makes it easier to obtain a comprehensive view of each gene. Taking CYP 9 J53 as an example (Fig. 2), users can input many types of keywords, e.g. “KFB49800.1”, “CYP 9 J53” or “9 J53” as search content. Pressing on the “GeneID” button will display the detailed information for this gene. At the top part of the gene information page, the users can view some fundamental information about CYP 9 J53, such as description, length and location. Clicking on the right “JBrowse” button enables users to visualize CYP 9 J53 under the background of the scaffold. Below these, the sequence information of CYP 9 J53 is presented. The exon regions are highlighted in red, and the remaining sequences are introns. Clicking on the “show pep” button allows amino acid sequences to be displayed. The lower portion of the information page is the functional feature description of CYP 9 J53, including orthologs, GO and KEGG pathways. In the ortholog part, many-one or many-many orthologous genes to the *An. sinensis* CYP 9 J53 in other mosquito species and fruit fly are displayed. There are links to get the sequences of the orthologs of *An. sinensis* genes in VectorBase and FlyBase. The prediction of GO terms shows that CYP 9 J53 belongs to “iron ion binding” (0005506), “electron carrier activity” (0009055), “heme binding” (0020037) and “oxidation-reduction process” (0055114) categories. Users can click the GO term for detailed term information. CYP 9 J53 participates in “Linoleic acid metabolism” pathway. Clicking the KO (ko00591) will open a new page to show the reference pathway map. Transcriptional results are shown at the bottom of the results page and include technology, comparison, regulation, fold change, published articles, source as a whole. Moreover, users can perform BLAST to find the best hit for the gene of interest via copy-paste sequences or upload sequence FASTA file. Different parameters can be reset to filter and parse the results again. Click one of the links in Score field will locate the pair-wise alignment between the query sequence and subject sequence.

### Download

ASGDB provides bulk data downloads, including assembled genome sequences, nucleotide sequences of putative genes, gene annotation and amino acid sequences. All data are hosted and accessible publicly via a browser directly. Meanwhile, manual curation of literature related to *An. sinensis* is carried out to fulfil the increasing research demands. We have collected *An. sinensis*-related English literature from PubMed (http://www.ncbi.nlm.nih.gov/pubmed/) and Chinese literature from CNKI (http://www.cnki.net/), Wanfang (http://www.wanfangdata.com.cn/) and VIP (http://www.cqvip.com/). The general information is organized as formatted lists, including title, authors, journal, year and volume. ASGDB also offers some full-text article links of English literature.

### Insecticide resistance-related genes

ASGDB provides the identification and classification information of detoxification enzyme superfamilies and cuticular proteins from *An. sinensis*. Three detoxification enzyme superfamilies (P450s, GSTs and CCEs) are primarily responsible for metabolic resistance in mosquitoes [49]. The cuticle is a major route of insecticide penetration, thickening or changing in the chemical composition of the cuticle serve as another resistance mechanism [50]. Although cuticular resistance has not yet been fully characterized at the molecular level, several examples of putative *cp*s that are the primary components of insect cuticle have been identified as the potential players in insecticide resistance [51–54].

On the basis of a literature review and in-depth data analysis, ASGDB provides detoxification enzyme and *cp* information from several mosquito species and the fruit fly *Drosophila melanogaster*, allowing users to perform rapid and convenient comparative analyses among different Diptera insect species. To aid insecticide resistance research, these genes are further classified into four P450 clans, seven *gst* classes, ten *cce* clades and eleven *cp* families.

### Resistance surveillance

The current release of ASGDB has recorded 551 insecticide-resistant phenotypic and genotypic events in *An. sinensis* in China. So far, the geographical distributions of the data cover the majority of *An. sinensis* distribution areas in China.

**Fig. 3:**
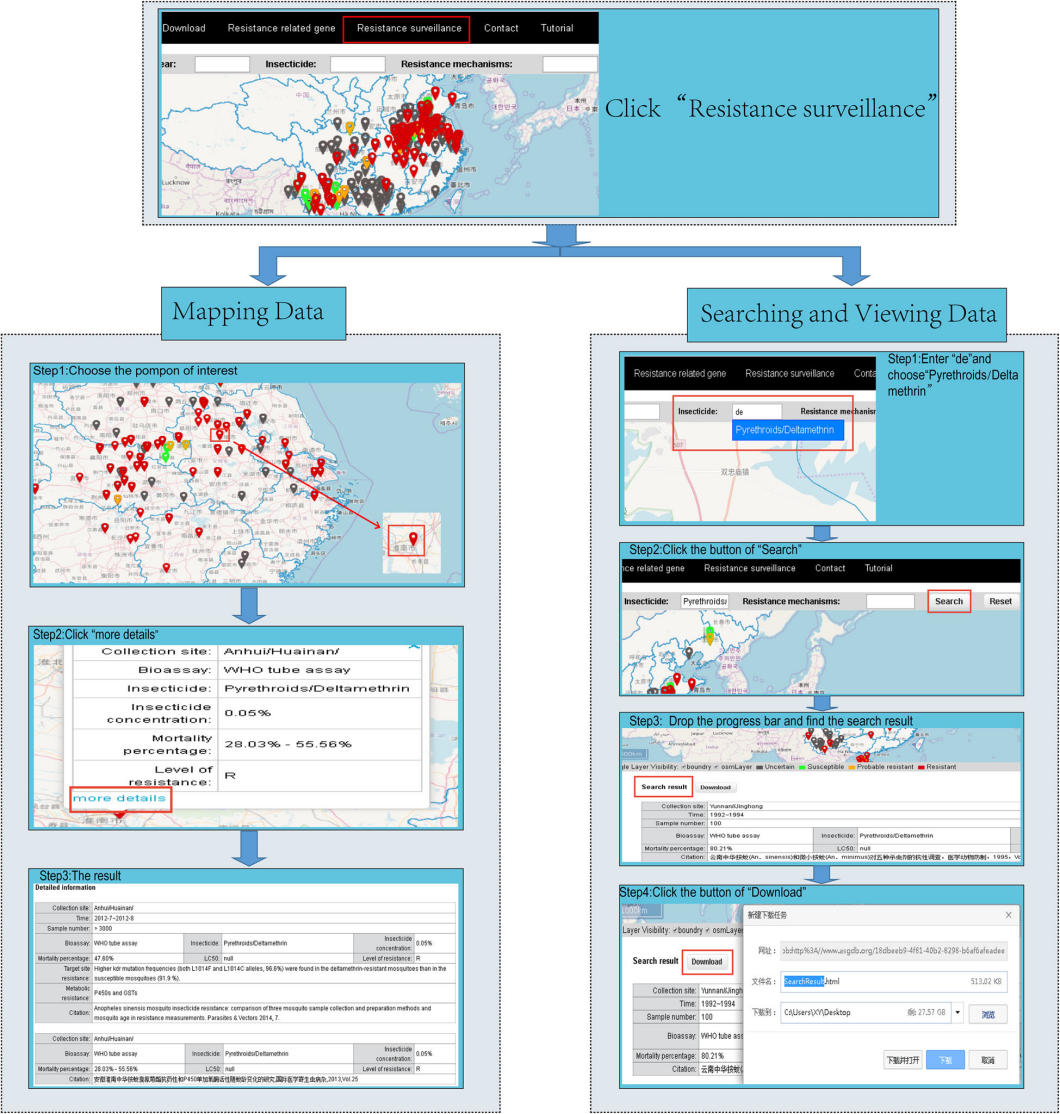
Screenshot showing how to search and retrieve insecticide-resistant related data on “Resistance surveillance” page

There are two ways to retrieve insecticide-resistant information in the database (Fig. 3). ASGDB provides an OpenLayers map-based interface for users to obtain data. All the mosquito-sampling sites are identified with small pompons. By clicking on the pompon, a pop-up text box will appear on the map with the most recent insecticide-resistant related record. We choose different colours for pompons to indicate insecticide-resistant levels (Grey: Uncertain; Green: Susceptible; Yellow: Probable resistant; Red: Resistant). Users can also browse all the relevant information in this region on the same page just below the map. It should be noted that when different sampling sites are in proximity, the red pompons might be very near or even superposed on each other. To avoid clicking on the non-target pompon, the users can zoom in to magnify their view of the map by tapping the “plus” button in the upper left corner or by scrolling up with mouse’s scroll wheel. Double left click on the map can also simultaneously centre the map and zoom in at the position clicked. Once users think they are done, they can use the “minus” button or scroll down with mouse’s scroll wheel to zoom out. To move the map, users can click and drag at any point on the map. ASGDB also provides the users with a search engine to facilitate obtaining the information of interest, such as collection site, collection year, insecticide and resistance mechanism. For example, if the users want to perform an insecticide based search using “deltamethrin”, they can type two letters “de” and a list of all possible matching terms will appear. Users can choose the requested term and click on the “Search” button, and only the matching pompons will appear on the map, and all records related to deltamethrin resistance will appear below the map. The retrieved information can be downloaded in bulk.

The data were manually extracted and concisely presented in three aspects: “Collection details”, “Insecticide resistance monitoring” and “Insecticide resistance mechanisms”. “Collection details” provides temporal and spatial information regarding sample collection. “Insecticide resistance monitoring” surveys the insecticide resistance status in the field population under different insecticide selection pressures. “Insecticide resistance mechanisms” provides further investigations of the resistance mechanisms, including the analysis of gene expression changes, *kdr* or *ace-1* mutations, or elevated enzyme activities of P450s, GSTs and CCEs. We also provided the citation of sources.

Users are encouraged to share their job data related to the insecticide resistance by using the submission procedure, which can ease the process of data collection and sharing, and benefit the dispersal of knowledge. In addition to relevant data, the participants also need to provide valid and open personal information, including institution and e-mail addresses. Although time consuming, it will not only improve content reliability but also increase users’ collaborations and communications.

### Contact

To establish a platform for community integration of *An. sinensis* data and to aid efficient management of knowledge on *An. sinensis*, ASGDB requires more participation in knowledge curation. Researchers can submit any comments, suggestions, or questions regarding all aspects of ASGDB. To promote researchers’ sharing and exchange of knowledge and ideas, the submission process is simple. No registration requirement is imposed, although users need to provide a valid email address so that our team can contact them in case of any queries.

### Tutorial

To help facilitate access and utilize these data, a general tutorial is also available in ASGDB. It provides schematic overview and demonstrates how to get started and navigate through the main features of ASGDB.

## Discussion

The basic objective of the ASGDB is to provide an integrative and comparative genomic resource particular to *An. sinensis*. It gathers a wide variety of genetic information, such as the whole draft genome sequences, annotation, pathway, GO terms, orthologous relationships and differentially expressed. The diverse data integration makes it possible to display correlations among various genetic factors and thus to will help users obtain genetic information faster and more accurately, which is a fundamental step in exploring valuable information for further study. As a one-stop resource platform, ASGDB contains a user-friendly interface, convenient search options and enhanced visualization tools, all of which make it easy for researchers to access and analyse whole-genome genomic data and information for *An. sinensis*, even those with little knowledge of bioinformatics.

Another aim of ASGDB is to share insecticide resistance information for *An. sinensis*. We collect resistance-related gene information in *An. sinensis* and other species. This genome-level data-mining strategy of resistance-related genes will also be useful for the follow-up functional study of resistance. For example, researchers could conveniently choose one specific gene of interest or a group of genes (e.g. by classification or gene-expanded clusters) to investigate how resistance phenotypes are generated. The information for genes differentially expressed between deltamethrin-susceptible and deltamethrin-resistant strains should also help identify candidate genes of interest. On the basis of data from the published literature, ASGDB provides a panoramic view of current insecticide-resistant studies of *An. sinensis* in China. These data cover 19 provinces and municipalities in China region, which vary substantially in geography, and economic and social environment. The records were initiated in the mid-1980s and continue to this day. These records systematically and continuously track the dynamics of the insecticide-resistant phenotypic and genotypic information, providing invaluable information to help us understand how insecticide resistance occurs and spreads in *An. sinensis* temporally and spatially. The excessive use of agricultural insecticides should be slowed before the occurrence of high resistance; therefore, the phenotypic information will be useful to adjust the types and concentrations of insecticides in a rotation scheme to fit local environmental conditions. It could promote the existing resistance management strategies to prolong the effectiveness of insecticides and prevent the occurrence of resistance. The evolution of insecticide resistance is conferred through complex mechanisms, typically requiring the interaction of multiple genes [55–58]. The genotypic data in ASGDB could provide clues to explain the molecular mechanism of insecticide resistance systematically. For example, we could observe whether target-site and metabolic resistance mechanisms occur singly or simultaneously, or judge which mechanism plays more important role in *An. sinensis* at different insecticide-resistance levels. The information of insecticide resistance at the mechanistic level, combined with results of bioassays, could also assist in providing powerful molecular diagnostic tools to aid the monitoring of insecticide resistance in *An. sinensis* at an early stage.

With increasing research on *An. sinensis*, genome re-sequencing, transcriptomic, proteomic and other omics studies are expected to grow continuously, especially with the development of sequencing technologies in the next few years. ASGDB will integrate more types of data and will be updated periodically to fulfil the growing research needs in addressing the genetic complexity of *An. sinensis*. Meanwhile, more insecticide resistance information from neighbouring countries will be integrated into ASGDB in the next step. Also, we encourage researchers to share insecticide-resistant data with the whole scientific community. To attract more participation from the scientific community for ASGDB and to make it an important platform for the insecticide resistance studies of *An. sinensis*, we will develop an incentive system to reward participants according to their contributions.

## Conclusions

To further extend our understanding of resistance mechanism and facilitate the implementation of resistance management strategies for mosquito vector control programs, a bioinformatics database named ASGDB was developed. High-quality draft genome sequence integrated with insecticide resistance-related literature, ASGDB provides (i) *An. sinensis* genome database; (ii) insecticide resistance-related gene data; and (iii) the insecticide resistance phenotyping and genotyping data in *An. sinensis* in China region. ASGDB was built to help users to mine data from the genome sequence of *An. sinensis* easily and effectively, especially with its advantages in insecticide resistance surveillance and control. The resistance surveillance related information collected in ASGDB provides dynamic and evolutionary resistance records in whole China region. Detailed records of insecticide resistance status of *An. sinensis* are useful for adjusting the types of insecticides in a rotation scheme to fit local environmental conditions.

## Additional file

Additional file 1: Table S1. The major classes and types of insecticides. (PDF 34 kb)

## Abbreviations

ASGDB: *Anopheles sinensis* genome database
BLAST: Basic Local Alignment Search Tool
CCE: Choline/carboxylesterase
CP: Cuticular protein
CSS: Cascading style sheets
GO: Gene ontology
GST: Glutathione S-transferase
ID: Identification
IRS: Indoor residual spraying
KEGG: Kyoto Encyclopedia of Genes and Genomes
KO: KEGG orthology
LLINs: Long-lasting insecticide-treated bednets
NCBI: National Center for Biotechnology Information
P450: Cytochrome P450
