# Supplementary material for: ASGDB: a specialised genomic resource for interpreting Anopheles sinensis insecticide resistance
Source: Parasit Vectors. 2018 Jan 10;11:32. doi: 10.1186/s13071-017-2584-8 (PMC5763776; doi:10.1186/s13071-017-2584-8)
Supplement: Additional file 1: Table S1. — The major classes and types of insecticides. (PDF 34 kb) [file 13071_2017_2584_MOESM1_ESM.pdf]

Table S1 The major classes and types of insecticides.

Classes	Types
Organochlorides	Camphechlor
	Chlordane
	DDT
	Endosulfan
	HCH,BHC
	lindane
Organophosphates	Azinphos-methyl
	Chlorpyrifos
	Diazinon
	Dichlorvos
	Dimethoate
	Ethephon
	Fenitrothion
	Phoxim
	Malathion
	Methamidophos
	Naled
	Omethoate
	Oxydemeton-methyl
	Trichlorfon
Carbamates	Aldicarb
	Alanycarb
	Aminocarb
	Bendiocarb
	Carbanolate
	Carbaryl
	Carbofuran
	Carbosulfan
	Dioxacarb
	Ethiofencarbo
	Fenobucarb
	Furadan
	Methomyl
	Pirimicarb
	Propoxur
	Thiodicarb
	Thiofanox
Pyrethroids	Allethrin
	Bifenthrin
	Cyfluthrin
	Cypermethrin
	Cyphenothrin

	Deltamethrin
	Esfenvalerate
	Etofenprox
	Fenpropathrin
	Fenvalerate
	Fenpropathrin
	Flucythrinate
	Flumethrin
	Imiprothrin
	Lambadacyhalothrin
	metofluthrin
	Permethrin
	Prallethrin
	Resmethrin
	Silafluofen
	Sumithrin
	Tau-fluvalinate
	Tefluthrin
	Tetramethrin
	Tralomethrin
	Transfluthrin
Neonicotinoids	Acetaniprid
	Clothianidin
	Dinotefuran
	Imidacloprid
	Nitenpyram
	Thiamethoxam
	Thiacloprid
